# Discrete-Time Quantum Walk with Phase Disorder: Localization and Entanglement Entropy

**DOI:** 10.1038/s41598-017-12077-0

**Published:** 2017-09-20

**Authors:** Meng Zeng, Ee Hou Yong

**Affiliations:** Division of Physics and Applied Physics, School of Physical and Mathematical Sciences, Nanyang Technological University, Singapore, 637371 Singapore

## Abstract

Quantum Walk (QW) has very different transport properties to its classical counterpart due to interference effects. Here we study the discrete-time quantum walk (DTQW) with on-site static/dynamic phase disorder following either binary or uniform distribution in both one and two dimensions. For one dimension, we consider the Hadamard coin; for two dimensions, we consider either a 2-level Hadamard coin (Hadamard walk) or a 4-level Grover coin (Grover walk) for the rotation in coin-space. We study the transport properties e.g. inverse participation ratio (IPR) and the standard deviation of the density function (*σ*) as well as the coin-position entanglement entropy (EE), due to the two types of phase disorders and the two types of coins. Our numerical simulations show that the dimensionality, the type of coins, and whether the disorder is static or dynamic play a pivotal role and lead to interesting behaviors of the DTQW. The distribution of the phase disorder has very minor effects on the quantum walk.

## Introduction

First conceived by Aharonov *et al*.^1^ in 1993 as a generalization of classical random walks, Quantum Walks (QWs) have since been extensively studied for possible applications in both computer science, physics, and even biology^2–4^. It differs dramatically from its classical counterpart and one of the most striking feature of standard QWs is that the probability distribution of the quantum particle spreads ballistically due to the existence of interference effects. This is in stark contrast to the diffusive nature of classical random walk. Due to the ballistic spreading of the density function, QWs have exponentially faster hitting times compared to the classical case and thus is a promising tool in quantum search algorithms and quantum information processing^5–7^. The readers are advised to refer to^8^ for a comprehensive review on this topic. Generally, there are two types of QWs: the discrete-time quantum walk (DTQW), which is the focus of our work, and the continuous-time quantum walk (CTQW)^9–11^. The two types of walk are shown to be related to each other through certain limit procedures^12,13^.

QWs have been experimentally realized using ultracold atoms in optical lattices and can be very useful to simulate strongly correlated many-body quantum systems^14–17^. Photonic systems have also been fruitful platforms for realizing QWs, in this case, the quantum walker are the photons^18,19^. DTQW is also a powerful tool for exploring topological phases in the sense that it can be used to realize all the classified topological phases in 1D and 2D with appropriately chosen and easily controllable QW protocol^20–22^. Wave localizations in different physical systems have been extensively studied since the seminal work of Anderson in 1958^23^. The fact that the quantum particle spreads ballistically motivated people to think about what happens to the transport properties when disorders are introduced. Localization effects in disordered QWs have been extensively investigated in recent years. There are two major types of disorders in QWs that are widely studied. One is disorder in the coin operation, i.e. the coin rotation angle is taken to be some random variables drawn from some probability distribution^24,25^. The other type of disordered QWs are those with on-site phase disorders, where the quantum particle will pick up some phase when it hops into certain sites of the lattice. The phase angles, like the coin rotation angles, can also be drawn from probability distributions, or it can simply be a single phase defect in the lattice^26–28^.

Recently, experiments have also been done to demonstrate ballistic spreading to localization cross-over in 1*D* ^29^ using light wave packets in photonic systems. Besides the effects of disorders on transport properties of QWs, another interesting quantity to look at is the coin-position entanglement, which is characterized by entanglement entropy (EE) given by the standard von Neumann entropy. The EE has been extensively studied for different 1*D* QW systems^30,31^. However, generalizations to higher dimensions are rarely seen^32^, especially when disorders are introduced. In this work we extensively study the 1*D* and 2*D* Hadamard walk and 2*D* Grover walk with random on-site phase disorders. Two type of phase disorders, one obeying binary distribution and the other obeying uniform distribution, are investigated in order to look at the possible effects of different random distributions on the behaviors of the QW. The physical quantities we are interested in are the inverse participation ratio (IPR), the width of the wave packet *σ* and the coin-position EE *S*_*E*_. It is demonstrated that the differences in effects due to the binarily distributed phase and the uniformly distributed phase are very minor. Instead, it is whether disorder is dynamic or static, the dimensionality of the system and the type of coin used that are playing the dominant role here. Each of these factors can change the behavior of the QW in a drastic and yet interesting way. For the results section, the first part is for the 1*D* Hadamard walk, the second part is for the 2*D* Hadamard walk, and the third part is for the 2*D* Grover walk. While presenting the results, comparisons between the different cases are made. For the Methods section, we present some optimized algorithm for accelerating the simulation.

## Results

### 1*D* Hadamard walk

For the 1*D* Hadamard walk with on-site phase disorder, the translation operator of the walk is given by

1 $$T=|\uparrow \rangle \langle \uparrow |\otimes \sum _{n}|n+1\rangle \langle n|{e}^{i{\varphi }_{n+1}^{t}}+|\downarrow \rangle \langle \downarrow |\otimes \sum _{n}|n-1\rangle \langle n|{e}^{i{\varphi }_{n-1}^{t}},$$

where |↑/↓〉〈↑/↓| is the spin projection operator for right/left-moving state and |*n*〉 denotes the position in the lattice. The coin operator is taken to be the standard Hadamard coin $$C=\frac{1}{\sqrt{2}}[\begin{array}{cc}1 & 1\\ 1 & -1\end{array}]$$.

In our case, we study two types of phase disorders, one with binary distribution and the other with uniform distribution. For binary phase disorder, ${\varphi }_{n}^{t}\in \{\alpha ,\beta \}$, meaning that the phase angle *ϕ* is chosen randomly from *α* and *β* with *α*, *β* ∈ [2, 2π). The phase angle can be position-dependent and time-dependent.In this work we investigate both the static disorder case and the dynamic disorder case. The transport properties of the QW only depends on the difference between the phases ${\rm{\Delta }}=|\alpha -\beta |$. Even though the phase angles are randomly chosen between *α* and *β* a global phase can always be factored out, be it *e* ^*iα*^ or *e* ^*iβ*^, and thus only the difference matters for physical observables. Effectively, the on-site random phase angles follow the Bernoulli distribution {0, Δ} with *p* = 0.5. For uniform phase distribution, we choose the phase interval to be from 0 to Δ in correspondence with the binary disorder.

We use the standard deviation to characterize the width of the probability distribution, given by

2 $$\sigma (t)=\sqrt{\sum _{m}{m}^{2}{P}_{m}(t)-{(\sum _{m}m{P}_{m}(t))}^{2}},$$

where *P*_*m*_(*t*) is the probability for the particle to be found at site *m* at time *t*, given by ${P}_{m}(t)=|{a}_{m}(t{)|}^{2}+|{b}_{m}(t{)|}^{2}$, with (*a*_*m*_(*t*), *b*_*m*_(*t*))^*T*^ being the two-component spinor of the quantum walker state at site *m* at time *t*. Because the QW considered in our case is symmetric, we do not have to worry about the shift of the center of the probability distribution. In order to look at the localization properties of the QW, we also calculated the inverse participation ratio (IPR), defined as

3 $${\rm{IPR}}(t)=\frac{1}{\sum _{m}{({P}_{m}(t))}^{2}}\mathrm{.}$$

The IPR gives the average number of lattice sites that the wave packet is spread over. Another quantity we look at is the coin-position EE *S*_*E*_. The coined QW is interesting because the coin state and the particle position are entangled and thus the coin operation at each step directly controls how the quantum particle moves on the next step and consequently controls the interference pattern of the quantum particle. The EE between the coin state and the particle position *S*_*E*_ follows the standard definition of von Neumann entropy for a bipartite pure state and it is calculated as

4 $${S}_{E}=-{\rm{tr}}({\rho }_{c}{\mathrm{log}}_{2}{\rho }_{c}),$$

where *ρ*_*c*_ is the reduced density matrix obtained by tracing over the the position basis the full density matrix *ρ* = |Ψ〉〈Ψ| of the QW system. The most general quantum state at some arbitrary time *t* is

5 $$|{\rm{\Psi }}(t)\rangle =\sum _{n}({a}_{n}(t)|\uparrow \rangle +{b}_{n}(t)|\downarrow \rangle )\otimes |n\rangle \mathrm{.}$$

The time-dependent density matrix of the system is given by

6 $$\begin{array}{rcl}\rho (t) & = & |{\rm{\Psi }}(t)\rangle \langle {\rm{\Psi }}(t)|\\  & = & \sum _{n,n^{\prime} }({a}_{n}(t)|\uparrow \rangle +{b}_{n}(t)|\downarrow \rangle )({a}^{\ast }(t)\langle \uparrow |+{b}_{n^{\prime} }^{\ast }(t)\langle \downarrow |)\otimes |n\rangle \langle n^{\prime} |\mathrm{.}\end{array}$$

The reduced density matrix is

7 $${\rho }_{c}(t)=\sum _{m}\langle m|\rho (t)|m\rangle =\sum _{m}\,[\begin{array}{cc}|{a}_{m}(t{)|}^{2} & {a}_{m}(t){b}_{m}^{\ast }(t)\\ {a}_{m}^{\ast }(t){b}_{m}(t) & |{b}_{m}(t{)|}^{2}\end{array}],$$

with eigenvalues

8 $${{\rm{\Lambda }}}_{\pm }=\frac{1}{2}\pm \frac{1}{2}\sqrt{1-4{\rm{\Gamma }}{\rm{\Omega }}+\mathrm{4|}\Pi {|}^{2}},$$

where ${\rm{\Gamma }}={\sum }_{m}|{a}_{m}(t{)|}^{2}$, ${\rm{\Omega }}={\sum }_{m}|{b}_{m}(t{)|}^{2}$, ${\rm{\Pi }}={\sum }_{m}{a}_{m}(t){b}_{m}^{\ast }(t)$ and Γ + Ω = 1. Consequently, the EE is explicitly given by

9 $${S}_{E}(t)=-{{\rm{\Lambda }}}_{+}{\mathrm{log}}_{2}{{\rm{\Lambda }}}_{+}-{{\rm{\Lambda }}}_{-}{\mathrm{log}}_{2}{{\rm{\Lambda }}}_{-}\mathrm{.}$$

The asymptotic value of the EE ${S}_{E}(t\to \infty )$ has been shown both numerically^33^ and analytically^34^ to be around 0.872 for disorder-free Hadamard walk with local initial conditions. The EE has also been investigated for QWs with charged particle in magnetic field^35^ as well as QWs with disordered coin operation^36,37^. Enhancement of EE in the presence of dynamic disorder have been demonstrated^36,37^. This is counter-intuitive because one would expect that dynamic disorder leads to quantum decoherence and thus the EE would be smaller compared to the disorder-free case.

**Figure 1:**
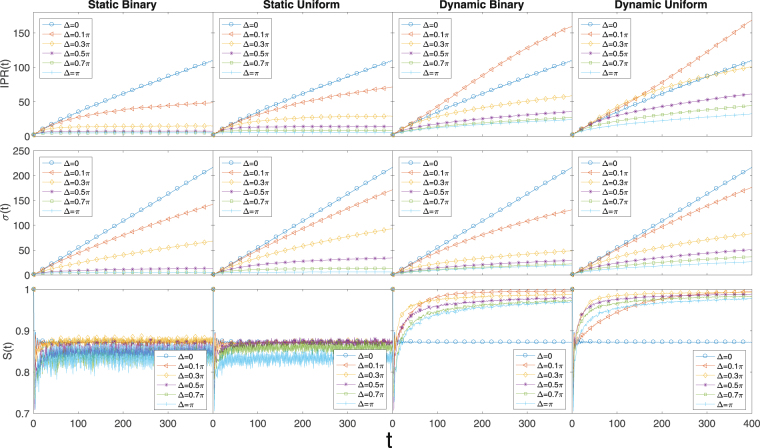
1*D* Hadamard walk with static/dynamic binary/uniform phase disorder. Binary and uniform phase disorders are positioned together to show the very minor difference in effects due to the two different types of random phase distributions. However, whether the random phase is static or dynamic does make a difference. Static disorder in the long time limit always reduces the EE and can lead to localization when the disorder strength is large enough, whereas dynamic disorder always enhances the EE and no localization occurs. The averages are calculated over 500 disorder realizations. Each subplot shown has 400 time steps.

Figure 1 summarizes the behaviors of IPR, *σ* and *S*_*E*_ under time evolution for static/dynamic binary/uniform phase disorders. Comparing the binary disorder column and the uniform disorder column, it can be seen that binary phase disorder and uniform phase disorder have very similar effects on the properties of the 1*D* QW. This is further confirmed when we generalize the model to 2*D* in later sections. We can see from Fig. 1 that when the phase disorder is static the 1*D* QW goes from ballistic spreading to complete localization as the disorder strength is tuned from 0 to *π*. It can be seen from the plots that the QW is already well localized when Δ reaches 0.3*π* since both the IPR and *σ* become constants of time. On the contrary, when dynamic disorder is introduced the IPR and *σ* are always increasing and the QW can at most be diffusive without localization. For the EE, we can see from the last row of Fig. 1 that in the 1*D* case EE is always reduced by static disorder and enhanced by dynamic disorder, which agrees with the findings in ref. ^36^.

One interesting point to note is the correlation between IPR and *σ*. We use the word correlation because IPR and *σ* are indeed related to certain extend, but it is by no means an one-to-one correspondence. From the plots it can be seen that *σ* is always reduced when disorder is introduced and it does not matter whether the disorder is static or dynamic, whereas IPR is only always reduced by static disorder and can be even increased by a small amount of dynamic disorder compared to disorder-free case. It is not difficult to see the difference between IPR and *σ* based on their definitions. IPR shows the average number of sites the wave packet is spread over, and *σ* characterizes the width of the wave packet. Imagine we have a distribution with double narrow peaks far away from each other, the width of the distribution would be much larger than the IPR. Therefore, in this sense the IPR is a more reliable quantity to determine localization and delocalization.

**Figure 2:**
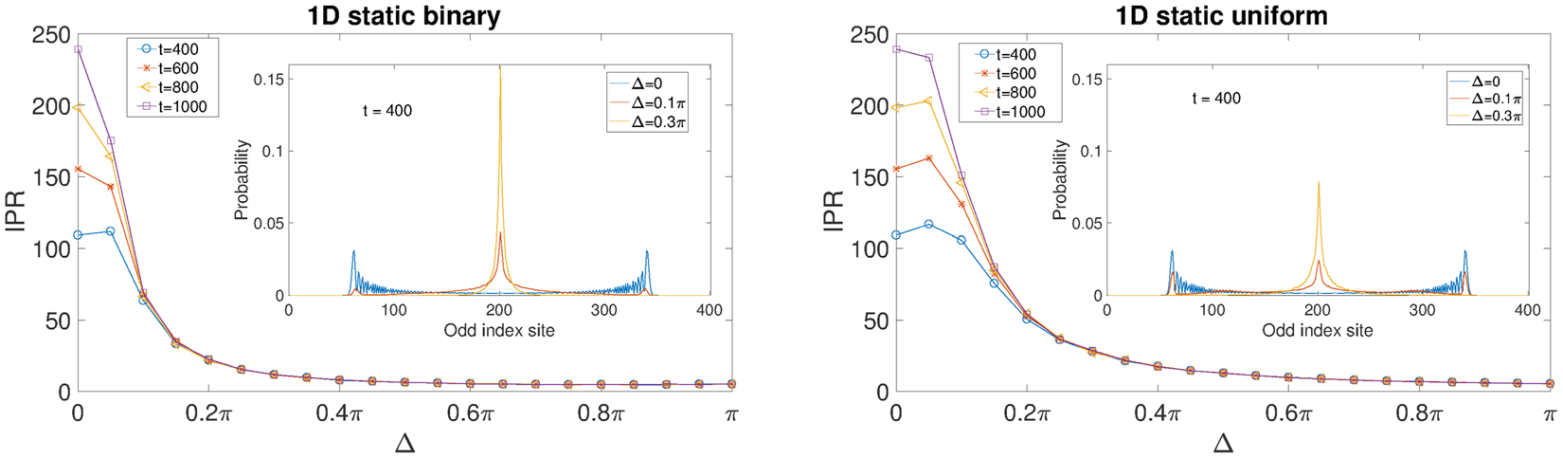
IPR as a function of the disorder strength Δ for four different time lengths of the 1*D* QW with static phase disorder. (**a**) Is for static binary disorder and (**b**) is for static uniform disorder. The insets show the probability distributions of the quantum walker at *t* = 400 when the disorder strength Δ = 0, 0.1*π*, 0.3*π* respectively. For the probability distribution only the odd-index sites are shown since all the even-index sites have zero probability. The averages are calculated over 500 disorder realizations.

Figure 2 plots the IPR of the 1*D* QW with static disorder to further illustrate the localization properties observed in Fig. 1. The subplot (a) is for static binary disorder and (b) is for static uniform disorder. Again we see very similar behaviors between the two. When the disorder strength is small, or more specifically when Δ is well below 0.2*π*, the four curves representing different walking time are well separated from each other, and when *t* increases from 400 to 1000 the IPR also keeps increasing, meaning the walk is not localized. However, when Δ goes beyond 0.2*π* the four curves start to collapse and hence the IPR stays almost the same when *t* increases, which indicates the onset of localization. Therefore, for either type of static disorder there will be a critical disorder strength Δ_*c*_ where the localization-delocalization transition happens. From Fig. 2 we can estimate that for static binary phase disorder Δ_*c*_ is close to 0.2*π* and for uniform static disorder Δ_*c*_ is close to 0.3*π*. The insets show the probability distribution of the quantum walker at *t* = 400 when Δ = 0, 0.1*π*, 0.3*π*. When there is no disorder the distribution has double peaks near the two ends. As the disorder strength is increased, the double peaks shrink and a peak starts to emerge at the center. When the disorder strength is increased further, the distribution will become a single localized peak at the center.

### 2*D* Hadamard walk

In this section, the previously studied 1*D* Hadamard walk is generalized to 2*D*. The quantum particle will pick up a phase, either static or dynamic, when moving in all the four directions. In the 2*D* case, we adopt the same quantum walk scheme as given in ref. ^35^, such that within each step there will be two Hadamard coin operations, one for moving in the *x* direction and the other for moving in the *y* direction. More specifically, the evolution operator is given by

10 $$U={T}_{y}(C\otimes I){T}_{x}(C\otimes I),$$

where *C* is usual Hadamard matrix used for the 1*D* case and *T*_*x*_, *T*_*y*_ are conditional translation operators containing the random phases given by

11 $$\begin{array}{rcl}{T}_{x} & = & |\uparrow \rangle \langle \uparrow |\otimes \sum _{m,n}|m+\mathrm{1,}\,n\rangle \langle m,n|{{\rm{e}}}^{i{\varphi }_{m+\mathrm{1,}n}^{t}}+|\downarrow \rangle \langle \downarrow |\otimes \sum _{m,n}|m-\mathrm{1,}\,n\rangle \langle m,\,n|{{\rm{e}}}^{i{\varphi }_{m-\mathrm{1,}n}^{t}},\\ {T}_{y} & = & |\uparrow \rangle \langle \uparrow |\otimes \sum _{m,n}|m,\,n+1\rangle \langle m,\,n|{{\rm{e}}}^{i{\varphi }_{m,n+1}^{t}}+|\downarrow \rangle \langle \downarrow |\otimes \sum _{m,n}|m,\,n-1\rangle \langle m,\,n|{{\rm{e}}}^{i{\varphi }_{m,n-1}^{t}}\mathrm{.}\end{array}$$

where ${\varphi }_{m,n}^{t}$ are the random phases that are possibly position-dependent and time-dependent. The width of the 2*D* probability distribution defined similarly with Eq. (2), as

12 $$\sigma (t)=\sqrt{\sum _{m,n}({m}^{2}+{n}^{2}){P}_{m,n}(t)-{(\sqrt{{m}^{2}+{n}^{2}}{P}_{m,n}(t))}^{2}},$$

**Figure 3:**
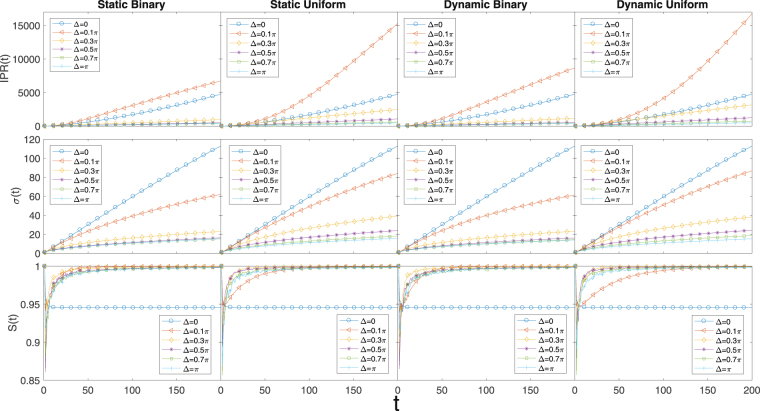
2*D* Hadamard walk with static/dynamic binary/uniform phase disorders. Again, similar to 1*D* Hadamard walk, there is no qualitative difference between binary and uniform disorders. The interesting thing here is that the 2*D* Hadamard walk is even insensitive to whether the disorder is static or dynamic. The asymptotic EE is always enhanced by both the static and dynamic disorder, and no localization occurs in this case. The averages are calculated over 50 disorder realizations. Each subplot shown has 200 time steps.

The reduced density matrix of the 2*D* QW system can be similarly calculated to be

13 $$\begin{array}{ccc}{\rho }_{c}(t) & = & \sum _{m,n}\langle m,n|\rho (t)|m,n\rangle \\  & = & [\begin{array}{cc}{\rm{\Gamma }} & {\rm{\Pi }}\\ {{\rm{\Pi }}}^{\ast } & {\rm{\Omega }}\end{array}],\end{array}$$

which is a straightforward generalization of Eq. (7). The matrix elements are now summations regarding the spinor components over two indices *m* and *n*. By doing the same calculation as Eqs (8) and (9), the EE in the 2*D* case can be calculated. The results for the 2*D* Hadamard walk are given in Fig. 3. Again we notice that the difference in asymptotic behaviors due to binary disorder and uniform disorder is very small except that the IPR increases faster for uniform disorder. A major difference between the 2*D* Hadamard walk and the 1*D* Hadamard walk is that in 2*D* the QW is almost insensitive to whether the disorder is static or dynamic, which is reflected in all the three quantities we look at. More specifically, based on Fig. 3, the asymptotic value of the EE is always enhanced by phase disorder and in fact tends to the maximum value of 1. The *σ* is always decreased by disorder, whereas the IPR can be increased by small amount of disorder (the *δ* = 0.1*π* curves in Fig. 3), all compared to the disorder-free case. No localization is observed based based on the IPR plots.

The long time limit of the eigenvalues of *ρ*_*c*_ in Eq. (13), and hence ${S}_{E}(t\to \infty )$, can be attributed to the asymptotic behaviors of Γ(*t*), Ω(*t*) and Π(*t*). The initial condition we started with for both the 1*D* and 2*D* walk is

14 $$|{\rm{\Psi }}(t=\mathrm{0)}\rangle =\mathrm{1/}\sqrt{2}(|\uparrow \rangle +i|\downarrow \rangle )\otimes |\overrightarrow{0}\rangle ,$$

which is symmetric in the spin-up component and the spin-down component. Under the Hadamard coin operation, the two components are treated symmetrically at each step of the walk. The phase disorder is completely random and thus after averaging over sufficiently number of ensembles in the long time limit, the quantum particle is equally likely to move towards the right and the left, and in the 2*D* case it will also be equally likely to move towards the up and down direction at each step of the walk. We know that whether the particle moves right or left and up or down is determined by the magnitudes of the two components of the spinor $({a}_{I},{b}_{I})$ with *I* being a collective index labeling the position of the particle on a lattice of any dimension. As a consequence, in the long time limit we should have ${\rm{\Gamma }}={\sum }_{I}|{a}_{I}{|}^{2}\to {\rm{\Omega }}={\sum }_{I}|{b}_{I}{|}^{2}$, and together with the normalization condition, we will have ${\rm{\Gamma }},{\rm{\Omega }}\to \mathrm{1/2}$. Therefore, based on Eq. 8, we can obtain

15 $$\mathop{lim}\limits_{t\to \infty }{{\rm{\Lambda }}}_{\pm }\to \frac{1}{2}\pm |{\rm{\Pi }}\mathrm{|.}$$

Thus, the EE given by Eq. (9) and also the one in the 2*D* case is asymptotically determined solely by the magnitude of the off-diagonal elements of the reduced density matrix. It is easy to see that from the entropy expression that the system is maximally entangled when $|{\rm{\Pi }}|\to 0$ and in fact ${S}_{E}(t\to \infty )$ is a monotonously decreasing when $|{\rm{\Pi }}|\in \mathrm{[0,}\,\mathrm{1/2)}$. In this limit, we have ${\rho }_{c}\propto {I}_{2}$, with *I*_2_ being the 2*D* identity matrix. In the language of quantum information, the total system is bipartite with one qubit |*s*〉∈*H*_2_ and the spatial degree of freedom $|{\overrightarrow{r}}_{I}\rangle \in {H}_{\infty }$ and the state of the QW system is a pure state $|{\rm{\Psi }}\rangle \in {H}_{2}\otimes {H}_{\infty }$. The von Neumann EE is only well-defined for a pure state and the pure state is maximally entangled when the reduced density matrix *ρ*_*c*_ is proportional to an identity matrix of the same dimension. The reduced density matrix takes a particularly simple form in the sense that it has the lowest possible dimension for an entangled quantum state due to the fact that a two-state coin is used. In general for a *N*-component spinor, the reduced density matrix will be *N* × *N* in dimension and will have slightly more complicated structures.

### 2*D* Grover walk

In this section, we further investigate the 2*D* QW using another type of coin, the standard 4-level Grover coin, to look at the different effects compared to the Hadamard coin studied in the previous section. The spin states of the quantum walker now become 4-component spinors, where each component controlling the movement into the four directions on the 2*D* square lattice. If we label the four mutually orthogonal spin states as |*u*〉, |*d*〉, |*l*〉, |*r*〉, representing moving up, down, left and right respectively, then the translation operator in this case would be given by

16 $$\begin{array}{rcl}T & = & |r\rangle \langle r|\otimes \sum _{m,n}|m+\mathrm{1,}\,n\rangle \langle m,n|{{\rm{e}}}^{i{\varphi }_{m+\mathrm{1,}n}^{t}}+|l\rangle \langle l|\otimes \sum _{m,n}|m-\mathrm{1,}\,n\rangle \langle m,n|{{\rm{e}}}^{i{\varphi }_{m-\mathrm{1,}n}^{t}}\\  &  & +|u\rangle \langle u|\otimes \sum _{m,n}|m,n+1\rangle \langle m,n|{{\rm{e}}}^{i{\varphi }_{m,n+1}^{t}}+|d\rangle \langle d|\otimes \sum _{m,n}|m,n-1\rangle \langle m,n|{{\rm{e}}}^{i{\varphi }_{m,n-1}^{t}}\mathrm{.}\end{array}$$

The Grover coin is given by

17 $$G=\frac{1}{2}[\begin{array}{cccc}-1 & 1 & 1 & 1\\ 1 & -1 & 1 & 1\\ 1 & 1 & -1 & 1\\ 1 & 1 & 1 & -1\end{array}].$$

Starting again with a symmetric initial spinor centered at the origin $|\Psi (t=\mathrm{0)}\rangle =\mathrm{1/2(1},1,1,{\mathrm{1)}}^{T}\otimes \mathrm{|0},0\rangle$, the general state of the walker at time *t* will be given by

18 $$\begin{array}{rcl}|{\rm{\Psi }}(t)\rangle  & = & TG|{\rm{\Psi }}(t=\mathrm{0)}\rangle \\  & = & \sum _{mn}{({a}_{mn}(t),{b}_{mn}(t),{c}_{mn}(t),{d}_{mn}(t))}^{T}\otimes |m,n\rangle \mathrm{.}\end{array}$$

**Figure 4:**
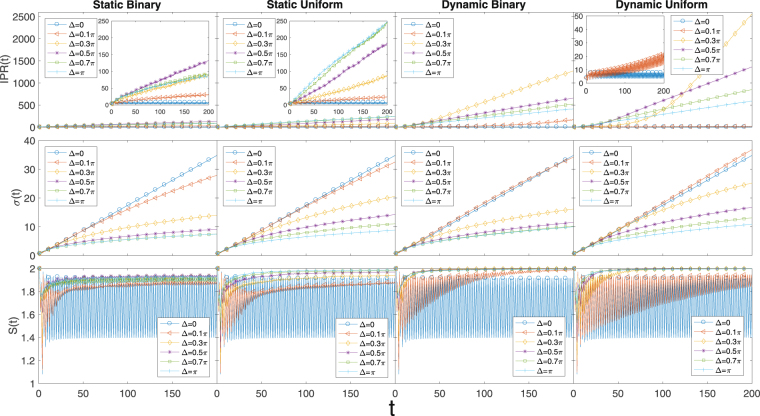
2*D* Grover walk with static/dynamic binary/uniform phase disorders. Again, similar to 1*D* and 2*D* Hadamard walks, there is no qualitative difference between binary and uniform disorders in the long time limit. However, there are some interesting major differences from the previous cases. Localization can happen for the Grover walk, but when there is no disorder. When disorder is introduced the IPR starts to increase with time (The insets for some of the IPR plots serve to show the details of the highly collapsed curves more clearly, the inset of the fourth panel of IPR plots only shows the amplified version of Δ = 0 and Δ = 0.1*π* since the other curves are easily distinguishable). When a small amount of dynamic disorder is introduced the width of the probability distribution can be increased even beyond the disorder free case, which does not happen for the Hadamard walks. The disorder-free EE for the Grover walk fluctuates indefinitely between two values, whereas for the Hadamard walks it approaches a well-defined limit. The averages are calculated over 50 disorder realizations. Each subplot shown has 200 time steps.

Then the probability distribution is given by ${P}_{mn}(t)=|{a}_{mn}(t{)|}^{2}+|{b}_{mn}(t{)|}^{2}+|{c}_{mn}(t{)|}^{2}+|{d}_{mn}(t{)|}^{2}$ and then the IPR and *σ* can be calculated in the same way as the 2*D* Hadamard walk. The reduced density matrix in this case will be 4 × 4 and is given by

19 $${\rho }_{c}=\sum _{mn}\,[\begin{array}{cccc}|{a}_{mn}{|}^{2} & {a}_{mn}{b}_{mn}^{\ast } & {a}_{mn}{c}_{mn}^{\ast } & {a}_{mn}{d}_{mn}^{\ast }\\ {b}_{mn}{a}_{mn}^{\ast } & |{b}_{mn}{|}^{2} & {b}_{mn}{c}_{mn}^{\ast } & {b}_{mn}{d}_{mn}^{\ast }\\ {c}_{mn}{a}_{mn}^{\ast } & {c}_{mn}{b}_{mn}^{\ast } & |{c}_{mn}{|}^{2} & {c}_{mn}{d}_{mn}^{\ast }\\ {d}_{mn}{a}_{mn}^{\ast } & {d}_{mn}{b}_{mn}^{\ast } & {d}_{mn}{c}_{mn}^{\ast } & |{d}_{mn}{|}^{2}\end{array}],$$

based on which the eigenvalues and thus the EE can be calculated for the 2*D* Grover walk. The results for the Grover walk are shown in Fig. 4. One interesting feature of the Grover walk is that when there is no disorder the IPR stays bounded to small values, even though the width of the wave packet increases ballistically, meaning that the wave packet is dynamically localized such that the quantum walker is concentrated on limited number of sites even though the concentrated parts of the distribution move further away over time to give a larger width. From the IPR plots, the wave packet spreads to more sites when disorder is introduced, with dynamic disorder being the more effective one. For better comparison between the different types of disorders, the same *y*− axis scale are used for different panels, resulting in severe collapse of some of the curves. Therefore, insets are put in to show the details more clearly for those highly collapsed curves. Another interesting feature of the Grover walk is that, very different from the previous Hadamard walks, the width of the wave packet can be larger than the disorder-free width when a small amount of dynamic disorder is added. This can be considered as an example of disorder-enhanced transport. However, when the disorder strength is increased further the walk becomes diffusive as usual.

The maximum value of the EE for the Grover walk now becomes 2 instead of 1 since the reduced density matrix is now 4 × 4. From the EE plots in Fig. 4, we can see the EE is generally enhanced by the disorders in the asymptotic limit. The disorder-free EE seems to fluctuate indefinitely between two values, around 1.4 and 1.9, whereas for the Hadamard walk in both 1*D* and 2*D* the EE approaches a constant.

## Conclusions

In this work, we extensively investigated the behavior of 1*D* and 2*D* Hadamard walk and 2*D* Grover walk with on-site random phase that are either binarily distributed or uniformly distributed. We find that the two types of random phase distributions have very minor effects on the general behavior of the QW in various cases. From our results the more dominating factors are whether the disorder is static or dynamic, the dimensionality of the QW system, and the types of coin used. For 1*D* Hadamard walk, localization can occur when the disorder is static and is strong enough. From the IPR and *σ* plots of Fig. 1 and the IPR plots in Fig. 2, we can see that both IPR and *σ* go to some constants when the number of steps is increased when Δ is well above 0.3*π*. This is a clear indication of localization of the wave packet around the starting point of the QW. The coin-position EE is always reduced by static disorder and enhanced by dynamic disorder in the long time limit. Quite different from the 1*D* Hadamard walk, the 2*D* Hadamard walk is almost insensitive to whether the phase disorder is static or dynamic. We do not see any qualitative difference between the different disorders based on Fig. 3. There is no localization in any case and the EE is always increased by the introduced disorder regardless of being static or dynamic.

For the 2*D* Grover walk, we see many interesting features not shared by the 1*D* or 2*D* Hadamard walk. There is dynamic localization when there is no disorder and the localization would be destroyed when disorder is introduced, in sharp contrast with the behavior of the Hadamard walks. Another interesting feature of the Grover walk is that the width of the wave packet increase faster than the disorder-free case when a small amount of dynamic disorder is introduced, which is quite counter-intuitive. The EE for the Grover walk behaves rather similarly with the 2*D* Hadamard walk, except that the disorder-free EE fluctuates indefinitely between two values instead of approaching a constant limit.

## Methods

In the numerical implementation of the static disorder, an array of random on-site phases are generated, whose length should match the maximum spread of the QW, and is unchanged until the designated number of steps are finished. This is considered as one realization of the QW. For dynamic disorder, the array of random phases is updated for each step of the QW. Thus the length of the array can be chosen to be the maximum spread of the QW, or it can be gradually increasing with the number of steps to save memory. In this work, the number of realizations used for calculating the IPR, *σ* and *S*_*E*_ is 500 for 1*D* and 50 for 2*D*. Parallel computation is recommended for the numerical simulation, especially the 2*D* case. When calculating these quantities as a function of the number of steps from 1 to *n*, one way of doing this is shown below. For each particular step *i*, the walk starts all over from the initial condition and repeats for the number of realizations.

$$\begin{array}{c}N\,{\rm{realizations}}\{\begin{array}{l}0\to 1\\ \mathrm{...}\quad \quad \quad \quad \quad \quad \quad {\rm{calculate}}\,{\rm{the}}\,{\rm{averages}}\,{\rm{for}}\,{\rm{the}}\,{{\rm{1}}}^{{\rm{st}}}\,{\rm{step}}\\ 0\to 1\end{array}\\ N\,{\rm{realizations}}\{\begin{array}{l}0\to 1\to 2\\ \mathrm{...}\quad \quad \quad \quad \quad \quad \quad {\rm{calculate}}\,{\rm{the}}\,{\rm{averages}}\,{\rm{for}}\,{\rm{the}}\,{{\rm{2}}}^{{\rm{nd}}}\,{\rm{step}}\\ 0\to 1\to 2\end{array}\\ \cdot \cdot \cdot \\ N\,{\rm{realizations}}\{\begin{array}{l}0\to 1\to \mathrm{2...}\to n\\ \mathrm{...}\quad \quad \quad \quad \quad \quad \quad {\rm{calculate}}\,{\rm{the}}\,{\rm{averages}}\,{\rm{for}}\,{\rm{the}}\,{n}\mbox{-}^{\mathrm{th}}\,{\rm{step}}\\ 0\to 1\to \mathrm{2...}\to n\end{array}\end{array}$$

However, we can do it much faster if we just do the following:

$$N\,{\rm{r}}{\rm{e}}{\rm{a}}{\rm{l}}{\rm{i}}{\rm{z}}{\rm{a}}{\rm{t}}{\rm{i}}{\rm{o}}{\rm{n}}{\rm{s}}\,\{\begin{array}{c}0\to 1\to \mathrm{2...}\to n\\ ...\\ 0\to 1\to \mathrm{2...}\to n\end{array}\,{\rm{c}}{\rm{a}}{\rm{l}}{\rm{c}}{\rm{u}}{\rm{l}}{\rm{a}}{\rm{t}}{\rm{e}}\,{\rm{t}}{\rm{h}}{\rm{e}}\,{\rm{q}}{\rm{u}}{\rm{a}}{\rm{n}}{\rm{t}}{\rm{i}}{\rm{t}}{\rm{i}}{\rm{e}}{\rm{s}}\,{\rm{a}}{\rm{t}}\,{\rm{e}}{\rm{a}}{\rm{c}}{\rm{h}}\,{\rm{s}}{\rm{t}}{\rm{e}}{\rm{p}}\,{\rm{w}}{\rm{i}}{\rm{t}}{\rm{h}}{\rm{i}}{\rm{n}}\,{\rm{e}}{\rm{a}}{\rm{c}}{\rm{h}}\,{\rm{r}}{\rm{e}}{\rm{a}}{\rm{l}}{\rm{i}}{\rm{z}}{\rm{a}}{\rm{t}}{\rm{i}}{\rm{o}}{\rm{n}}$$

where the quantities are calculated and stored for each step within each realization, after which the averages can be calculated at all the time steps using the stored values, without starting the walk all over again when doing the calculation for a different time step. With this algorithm, the simulation can be substantially accelerated.

Another thing we could do to optimize the code is to directly use closed forms of the eigenvalues for the reduced density matrix in order to calculate the EE because numerical diagonalization takes much time. For a 2 × 2 density matrix, this can be very easily done. For 4 × 4 matrices, the roots of the 4th order characteristic polynomial will be very complicated, but the closed forms do exist.
